# Assessment of clinical competence of graduating medical students and associated factors in Ethiopia

**DOI:** 10.1186/s12909-023-04939-1

**Published:** 2024-01-03

**Authors:** Daniel Dejene, Firew Ayalew, Tegbar Yigzaw, Alemseged Woretaw, Marco Versluis, Jelle Stekelenburg

**Affiliations:** 1 Department of Health Sciences, Global Health, University Medical Centre Groningen/University of Groningen, Groningen, Netherlands; 2 Jhpiego Ethiopia, P.O. Box:2881, code, 1250 Addis Ababa, Ethiopia

**Keywords:** Graduating medical students, Clinical competence, Ethiopia

## Abstract

**Background:**

Ethiopia has scaled up medical education to improve access to healthcare which presented challenges to maintaining training quality. We conducted a study to assess the clinical competence of graduating medical students and the associated factors.

**Methods and materials:**

A pretest assessment of a quasi-experimental study was conducted in 10 medical schools with a sample size of 240 students. We randomly selected 24 students per school. Clinical competence was assessed in a 12-station objective structured clinical examination. The clinical learning environment (CLE), simulation training, and practice exposure were self-rated. Mean scores for clinical competence, and satisfaction in the CLE and simulation training were calculated. Proportions of students with practice exposure, and who agreed on CLE and simulation items were done. Independent t-tests were used to look at competence differences among subgroups. Bivariate and multiple linear regression models were fitted for the outcome variable: competence score. A 95% statistical confidence interval and *p*-value < 0.05 were used for making statistical decisions. A 75% cut-off score was used to compare competence scores.

**Results:**

Graduating medical students had a mean competence score of 72%. Low scores were reported in performing manual vacuum aspiration (62%), lumbar puncture (64%), and managing childbirth (66%). Female students (73%) had a significantly higher competence score than males (70%). Higher cumulative grade point average (CGPA), positive appraisal of the CLE, and conducting more clinical procedures were associated with greater competence scores. Nearly half of the students were not satisfied with the clinical practice particularly due to the large student number and issues affecting the performance assessment. About two-thirds of the students were not satisfied with the sufficiency of models and equipment, and the quality of feedback during simulation training. Nearly one-third of the students never performed lumbar puncture, manual vacuum aspiration, and venipuncture.

**Conclusions:**

Medical students had suboptimal clinical competence. A better clinical learning environment, higher cumulative GPA, and more practice exposure are associated with higher scores. There is a need to improve student clinical practice and simulation training. Strengthening school accreditation and graduates’ licensing examinations is also a way forward.

## Background

Many countries across the world have serious health workforce challenges [1]. In 2020, a global shortage of 15.4 million health workers with huge skill mix imbalances and maldistributions was reported [2]. Sub-Saharan Africa (SSA) is disproportionally affected, with relatively few health workers and a high burden of disease [3, 4]. The density of physicians in SSA countries (< 0.3/1000 population in 2018) was very low as compared to the high-income countries (2.0–5.0) [5, 6].

Like many African countries, Ethiopia invested to increase the number of healthcare workers. It has been implementing the national human resources for health strategic plan which set a goal of increasing the stock of physicians by five-fold from 5411 in 2016 to 28,121 in 2025 [7]. As a result, medical schools have been expanded from 5 in 2005 to 43 in 2022, including 10 private colleges [8]. The annual number of graduates has increased tenfold and reached 1500 – 1600. Despite the positive strides, addressing the health workforce challenges in the country is far from finished. For instance, the density of physicians was still low, at 0.1 per 1000 people [9].

Increasing the number of training institutions can only improve population health when the quality of training is ensured. Workforce quality is an important part of the solutions to the global human resource crisis. Enrolment of so many students with limited medical school adaptations has fueled the quality issues in the country [10–12]. Despite the commendable efforts in expanding medical education, Ethiopia has lagged behind the WHO’s recommendations in reforming and implementing medical curricula, expanding student clinical sites and simulation-based training, and strengthening accreditation [13]. There have been shortages of learning resources and experienced faculty [14, 15]. With the rapid expansion, the training quality concerns have further deepened which might have affected student learning. Practice analysis of Junior physicians also reported substantial clinical skill gaps [16].

The effect of the training quality gaps on the competence of medical students is not well studied. Therefore, we conducted a study to assess the clinical competence of graduating medical students and associated factors.

## Methods and materials

### Study design and period

This pretest assessment is part of a quasi-experimental study aiming to assess the impact of project interventions in improving the quality of medical education in Ethiopia. Considering the complexity of the medical education environment for a random control study, we used a quasi-experimental design which is well suited to examine the cause-and-effect relationships among variables [17]. A posttest part of this quasi-experimental study will be repeated in 2025. Changes in the clinical competence and the associated factors from the pretest level will be used as evaluation measures. This pretest study was carried out in July and August 2022.

### Study setting and study population

There were 43 medical schools in Ethiopia, including 33 public and 10 private schools. Most medical schools admit high school graduates using a direct entry scheme while some accept graduates in the health and other science fields using a graduate entry scheme. The duration of medical education is 6 academic years, including 1 year of internship at the end. Starting from the third academic year, medical education is provided at hospitals and other clinical settings. The study population for this study was the 1556 undergraduate medical students who completed or were nearly completing their internship program and expected to graduate in 2022.

### Sample size and selection criteria

Ten schools that had graduating classes around the same period were selected in consultation with the Ethiopian Medical Association and the Ministry of Health. Of the 10 selected schools, four employed graduate entry schemes, and two were private schools. About 875 medical students were expected to graduate from the 10 schools in 2022. To determine the sample size, we considered a 95% confidence level, 80% statistical power, 1:1 optimal allocation (sample ratio of intervention to comparison), a moderate effect size of 0.5, and a design effect of two. We calculated the minimum sample size of 218 graduating students. After including a non-response rate of 10%, the final sample size was found to be 240.

### Sampling procedures

To include 240 students from the 10 medical schools, we considered recruiting 24 students per school. Because our study aimed at observing students’ competence using a 12-station objective structured clinical examination (OSCE), including 24 students ensured better competency observations and the attainment of adequate data points at each school. To develop the sampling frame, we requested the lists of graduating medical students from the deans’ offices. Using a lottery method, we randomly selected 24 students. We provided the lists of students to the assessors (data collectors) who invited students to participate in the study. If the selected students were not willing to participate for any reason, the data collectors thanked them and did not replace them.

### Measurements and instruments

The key variables of interest were clinical competence, clinical learning environment (CLE), simulation training, and practice exposure. We assessed clinical competence using a 12-station OSCE, a reliable method to assess clinical skills [18, 19]. Using global and national standards, OSCE case scenarios, assessment rubrics, and assessor instructions were developed [20–22]. The stations focused on the core competencies required for the provision of safe medical care which included 10 manned stations: taking a focused history, conducting pericardium examination, providing patient education and counseling for diabetes mellitus, conducting Leopold’s maneuver, conducting manual vacuum aspiration (MVA) for incomplete abortion, managing childbirth, performing wound suturing, taking emergency response for polytrauma, obtaining consent for hernia repair, and performing lumber puncture (LP). The remaining two stations were unmanned and focused on interpreting chest X-rays and complete blood cell counts for tuberculosis (TB) patients; and prescribing medication for a malaria case. To ensure content validity, we reviewed the stations with both subject matter experts and medical educators. The assessment rubrics had 4 to 7 items and followed a five-point global rating scale (GRS), where 1 meant “poor performance”, 2 “unsatisfactory performance”, 3 “satisfactory performance but not good”, 4 “good performance”, and 5 “excellent performance”. To assess the clinical learning environment, we used a validated clinical learning evaluation questionnaire (CLEQ) [23], a tool for measuring a learning climate in the clinical settings for undergraduate medical students with 18 items organized in four domains: clinical cases, motivation of students, supervision by preceptors, and clinical encounter organization. Similarly, we developed another 10-item structured tool to assess the quality of simulation training using guidelines and literature [24, 25]. Students self-assessed their experiences on each item of the two questionnaires on 5 points Likert scale, where 1 meant strongly disagree, 2 disagree, 3 neutral, 4 agree, and 5 strongly agree. In addition, we developed a structured tool to determine the practice exposure of students to 12 task procedures in the past 12 months. The list of procedures was adopted from the national scope of practice and training curricula. There were also variables about the background characteristics of medical students.

## Data collection

The OSCE was administered by 18 physicians and medical educators. A two-day training was given to assessors on data collection procedures, tools, ethical principles, data quality assurance, and the CommCare software application. Assessors informed study participants about the purpose, procedure, and ethical principles of the study and obtained consent. Study participants completed the CLEQ, simulation training quality, and practice exposure questionnaires. The study participants were encouraged to provide accurate information and/or the best plausible response to each item. For the OSCE stations, the data collectors made sure that all essential logistics (standardized patients, models, medical equipment, medical supplies, assessor instructions, and assessment rubrics) were available. The data collectors asked the study participants to undertake the required tasks at each OSCE station within 12 minutes. They conducted direct observations of students’ performance and rated them exclusively using the GRS. The data collectors were also closely supported by 6 supervisors to check errors and omissions. The OSCE assessment rubrics had a total of 64 items and an average Cronbach’s alpha value of 0.79 with a range of 0.62–0. 89 (Table 2).

### Data management and analysis

We exported the data from CommCare v. 2.53 to SPSS v. 27 for data cleaning and statistical analyses. Summary statistics were computed for all key variables to check outliers and missing data. Cronbach alpha coefficients were computed to assess the consistencies of items listed in each competence. Means, medians, standard deviations, proportions, tables, and graphs were computed. Mean scores for the 12 competencies and the overall composite mean score were computed. Mean satisfaction scores on CLE and simulation training were also calculated. To conduct desired statistical tests using continuous quantitative variables, we decided to merge many items of CLE and simulation into a single one by transforming the Likert scale data into composite mean scores [26]. The five-point Likert scale measures of CLE and simulation training were also grouped into two categories (strongly agree and agree as “agree”, and strongly disagree, disagree and neutral as” disagree”), and proportions were conducted to give a meaningful interpretation. Proportions were calculated for practice exposure. Since the curriculum did not specify thresholds for the number of clinical procedures expected to be performed, the median for each procedure was used as a cutoff point to decide between high and low exposures. The median values were used as measures of central tendency since the data had outlier values. An independent sample t-test was used to make comparisons between male and female students, private and public schools, direct and graduate entries, students with high and low clinical exposures, and students with high and low CGPA. We also checked the necessary assumptions for regression analysis and ensured the model’s adequacy [27]. Bivariate and multiple linear regression models were then fitted. The outcome variable was the competence score. The independent variables were age, sex, school type, cumulative grade point average (CGPA), school entry scheme, composite satisfaction scores for the four CLE domains, and simulation training. We considered all independent variables with *P* < 0.025 at the bivariate level for inclusion in the multivariable regression analysis. A 95% statistical confidence interval and *p*-value < 0.05 were used for making statistical significance decisions. Students are expected to master essential skills for safe and beginner-level healthcare delivery at the point of graduation. A 75% cut-off score which is recommended in mastery learning was used in comparing the competence scores [28].

### Data quality assurance

We adopted a standardized CLEQ data collection tool to assess the clinical learning environment. In the case of no standardized tools, the questionnaires for OSCE, simulation training, and practice exposure were reviewed and validated by medical education experts. We recruited senior medical education experts who have experience in conducting OSCE as data collectors. A two-day training was provided to data collectors to standardize data collection. Study investigators along with supervisors ensured that quality data were collected. We used an electronic data collection application to prevent data entry errors and supervised the data collection process.

### Ethics

Ethical approval for the study was obtained from the Ethiopian Public Health Association and Johns Hopkins Bloomberg School of Public Health Institutional Review Board with IRB number 21116. Permission to conduct the study was also obtained from the Ministry of Health (MOH) and the deans of medical schools. Study participants provided informed oral consent, and measures were taken to protect autonomy and data confidentiality.

## Results

### Background characteristics

A total of 218 graduating medical students took part in this study with a response rate of 90.8%. Their mean age was 27.1 years. The majority of study participants were males (70.2%), from public schools (86.2%), and had a cumulative grade point average (CGPA) of more than 3.00 at the beginning of the internship (74.5%). Graduates from private medical schools were younger (mean age 25.7 vs. 27.4 years), had higher proportions of female students (66.7% vs. 23.9%), and had higher CGPA (3.33 vs. 3.19) than those from public medical schools (Table 1).

**Table 1:** Background characteristics of study participants (*N* = 218)

Characteristics	Public school (*N* = 188)		Private school (*N* = 30)		Total (*N* = 218)	
	No	%	No	%	No	%
**Age in years**						
< 27 years	105	55.9	29	96.7	134	61.5
> =27 years	83	44.1	1	3.3	84	38.5
Mean age [SD] in years	27.4 [0.88]		25.4 [2.42]		27.1 [2.35]	
The age range (in years)	25–37		25–28		25–37	
**Sex**						
Male	143	76.1	10	33.3	153	70.2
Female	45	23.9	20	66.7	65	29.8
**Place of birth**						
Urban	127	67.6	28	93.3	155	71.1
Rural	59	31.4	2	6.7	63	28.9
**Student entry scheme**						
Direct entry scheme	99	52.7	29	96.7	128	58.7
Graduate entry scheme	89	47.3	1	3.3	90	41.3
**Medicine a first career choice**						
Yes	175	93.1	28	93.3	203	93.1
No	13	6.9	2	6.7	15	6.9
**CGPA at the start of the internship**						
> =3.50	44	23.4	13	43.3	57	26.1
3.00–3.49	93	49.5	13	43.3	106	48.6
< 3.00	51	27.1	4	13.3	55	25.2
Mean CGPA [range]	3.19 [2.4, 4.0]		3.33 [2.4, 3.8]		3.2 [2.4, 4.0]	

### Competence scores of graduating medical students

**Fig. 1:**
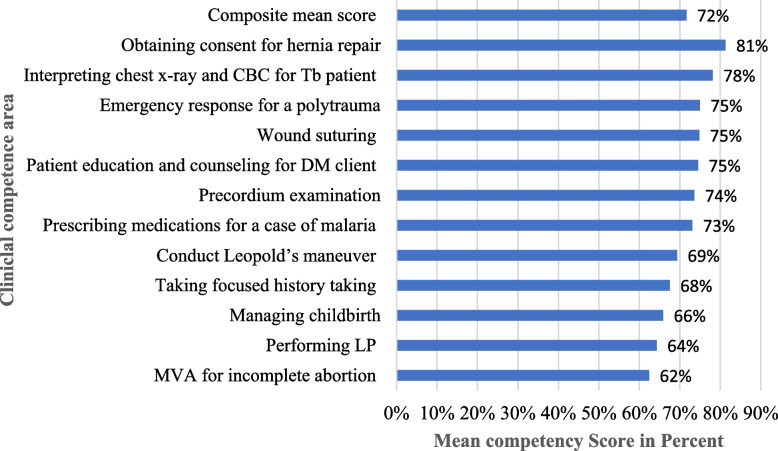
Mean clinical competece scores of graduating medical studetns in percentage

The overall mean competence score of graduating medical students was 72%. The highest scores were observed for obtaining consent for hernia repair (81%), and interpreting chest X-rays and CBC for TB patients (78%). On the other hand, competence scores were relatively low in conducting MVA for incomplete abortion (62%), performing LP (64%), and managing childbirth (66%) (Fig. 1).

There was no statistically significant difference in overall student competence scores between public (71.6) and private (71.7) schools. However, students from public schools had significantly better scores in taking a focused history (*p* = 0.001), conducting precordium examination (*p* = 0.002), and obtaining consent for hernia patients (*p* = < 0.001). On the other hand, students from private medical had significantly better scores in patient education and counseling (*p* = 0.03), prescribing medication for a malaria case (*p* < =0.001), and wound suturing (*p* = 0.02) (Table 2).

**Table 2:** Mean competence scores of study participants in percentage by school type

Skill station	Number of items	Cronbach’s alpha	Public school mean score, (SD) a	Private school mean score, (SD) b	Mean score difference (c = a - b)	*p*-value (t-test)
Focused history taking	5	0.78	69.2 (14.5)	56.8 (9.9)	12.4	**< 0.001**
Precordium examination	5	0.87	74.5 (16.5)	67.7 (9.5)	6.8	**0.002**
Interpreting chest x-ray & complete blood count for TB case	4	0.64	78.6 (13.7)	75.3 (15.5)	3.2	0.235
Patient education & counseling for DM case	7	0.89	73.7 (14.1)	79.7 (11.4)	−6.0	**0.03**
Prescribing medications for malaria case	4	0.62	71.2 (16.3)	85.0 (12.7)	−13.8	**< 0.001**
Conduct Leopold’s maneuver	6	0.81	70.0 (17.1)	65.0 (12.7)	5.0	0.126
MVA for incomplete abortion	7	0.86	61.8 (17.8)	66.2 (12.1)	−4.4	0.093
Managing childbirth	5	0.78	65.5 (16.0)	68.4 (17.4)	−2.9	0.361
Wound suturing	5	0.86	74.0 (17.7)	80.0 (11.6)	−6.0	**0.020**
Emergency response for a polytrauma	5	0.77	74.8 (13.6)	75.7 (15.5)	−0.9	0.759
Obtaining consent for hernia repair	5	0.77	82.8 (16.3)	71.9 (10.5)	10.9	**< 0.001**
Performing LP	6	0.87	63.5 (20.4)	69.0 (9.3)	−5.5	0.156
**Composite mean score**	**64**	**0.79**	**71.6 (7.80**	**71.7 (4.8)**	−0.1	0.928

The mean competence scores of the graduate entry students (72.3) and direct entry ones (71.2) had no significant difference. However, graduate entry students scored higher in precordium examination (p < 0.001), conducting Leopold’s maneuver (*p* < 0.001), and obtaining consent for hernia repair (*p* = 0.004). On the other hand, direct entry students performed better in tasks of patient education and counseling(*p* = 0.002) and lumbar puncture (*p* = 0.002) (Table 3)

**Table 3:** Mean competence scores of study participants in percentage by medical school entry schemes

Skill station	Direct mean score, (SD) a	Graduate mean score, (SD) b	Mean score difference c = a- b	*p*-value (t-test)
Focused history taking	66.8 (13.3)	68.6 (16.3	−1.8	0.380
Precordium examination	70.4 (15.2)	78.2 (15.8)	−7.8	**< 0.001**
Interpreting chest x-ray and complete blood count for TB case	79.8 (13.8)	75.8 (13.9)	4.0	0.040
Patient education & counseling for DM case	77.0 (13.20	71.0 (14.1)	6.0	**0.002**
Prescribing medications for malaria case	71.3(17.7)	75.7 (14.3)	−4.4	0.040
Conduct Leopold’s maneuver	64.4 (15.5)	76.3 (15.6)	−11.9	**< 0.001**
MVA for incomplete abortion	62.0 (17.1)	63.1 (17.3)	−1.1	0.650
Managing childbirth	67.4 (15.2)	63.7 (17.3)	3.7	0.100
Wound suturing	73.1 (18.2)	77.3 (15.3)	−4.2	0.070
Emergency response for a polytrauma	76.4 (14.4)	72.9 (12.8)	3.5	0.070
Obtaining consent for hernia repair	78.7 (16.2)	85.0 (15.1)	−6.3	**0.004**
Performing LP	67.7 (18.0)	59.4 (20.3)	8.3	**0.002**
**Composite mean score**	**71.2 (7.5)**	**72.3 (7.4)**	−1.1	0.317

Female medical students (73.2%) had a significantly higher competence score than their male counterparts (71.0%) (*p* = 0.04). Female students outperformed males in four skill areas: interpreting chest X-rays and CBC for TB patients (*p* = 0.02), prescribing medication for malaria cases (*p* = 0.04), conducting MVA for incomplete abortion (*p* = 0.02), and performing lumbar puncture (*p* = 0.01) (Table 4)

### Clinical learning environment

**Fig. 2:**
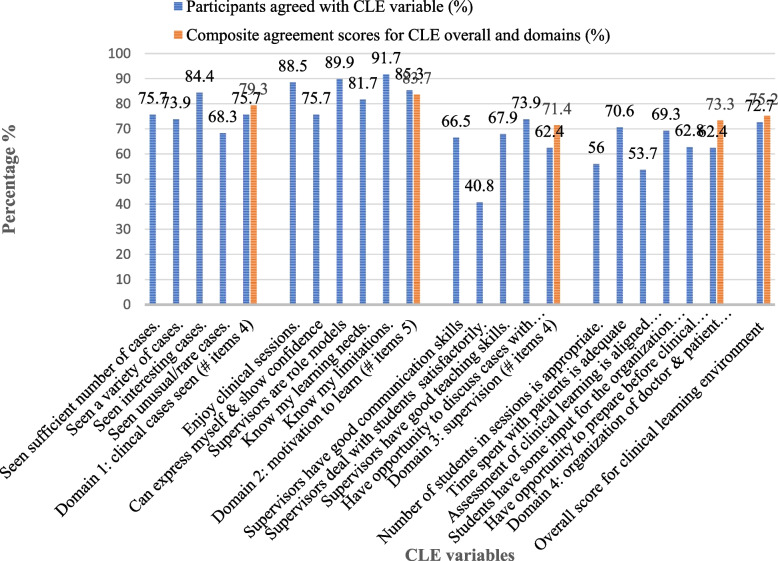
Percent of medical graduates who were satisfied with CLE items and satisfaction scores by CLE domain (*N* = 218)

Medical students had an overall mean CLE satisfaction score of 75.2%. The motivation of students during clinical practicum had the highest score (83.7%). In addition, the majority of the students knew their learning limitations (91.7%), enjoyed learning at clinical practice sites (88.5%), and thought that the supervisors were good role models (89.9%). However, supervision of students during practicum had a low score (71.4%). Significant of them also reported that the way the supervisors dealt with medical students was satisfactory (40.8%), the number of students in clinical sessions was appropriate (56.0%), and the assessment of clinical learning was aligned with objectives (53.7%) (Fig. 2).

**Table 4:** Mean competence scores of study participants in percentage by sex

Skill station	Male mean score (SD) a	Female mean score (SD) b	Mean score difference c = a-b	p-value (t-test)
Focused history taking	67.7 (14.9)	66.9 (13.9)	0.8	0.690
Precordium examination	74.5 (15.9)	71.4 (15.8)	3.1	0.180
Interpreting chest x-ray and CBC for TB case	76.7 (14.0)	81.5 (13.3)	−4.5	**0.020**
Patient education and counseling for DM case	72.6 (13.8)	79.0 (13.2)	− 6.4	0.830
Prescribing medications for a case of malaria	72.9 (16.1)	73.5 (17.6)	−0,6	**0.040**
Conduct Leopold’s maneuver	70.9 (16.8)	65.9 (15.8)	5.0	0.150
MVA for incomplete abortion	61.3 (17.4)	65.0 (16.5)	−3.7	**0.020**
Managing childbirth	64.2 (16.4)	69.9 (15.0)	−5.7	0.090
Wound suturing	73.5 (17.9)	77.9 (14.7)	−4.4	0.120
Emergency response for a polytrauma	74.0 (13.9)	77.2 (13.6)	−3.2	0.120
Obtaining consent for hernia repair	81.0 (17.0)	82.0 (13.6)	−1.0	0.660
Performing LP	62.3 (20.3)	68.9 (16.1)	−6.6	**0.010**
**Composite mean score**	**71.0 (7.4)**	**73.2 (7.3)**	−2.2	**0.040**

Medical students with a CGPA of 3.25 or more (74.3%) had a significantly higher competence score than those with a CGPA of 3.25 (69.5%) (*p* < 0.001). Those with a CGPA of at least 3.25 also outperformed in six competence areas: performing precordium examination (*p* = 0.024), interpreting chest X-rays and complete blood count for TB case (*p* = 0.006), conducting Leopold’s maneuver (*p* = 0.004), wound suturing (*p* < 0.001), emergency response for polytrauma (*p* = 0.006), and performing lumbar puncture (*p* = 0.007) (Table 5)

### Simulation training quality

**Fig. 3:**
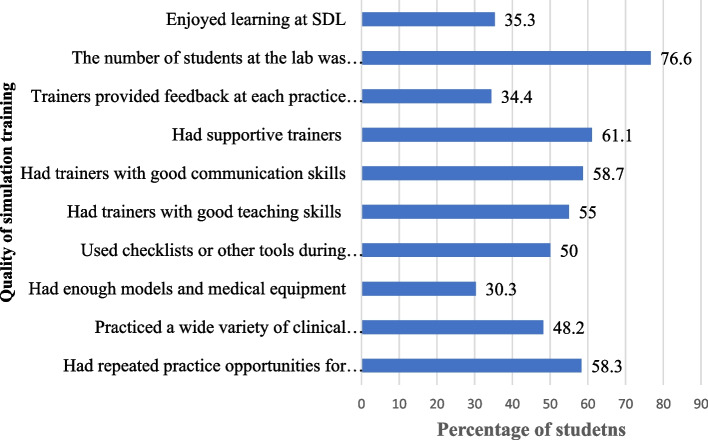
Percent of graduating medical students who were satisfied with the simulation training quality (*N* = 218)

Overall, 51% of participants were satisfied with the quality of simulation training. Specifically, 77% of respondents said the number of students at the skills development lab (SDL) was appropriate, and 61% acknowledged that supportive trainers were available. About two-thirds of the respondents expressed dissatisfaction with the availability of models and equipment at the lab, and the feedback provided at each practice session, and did not enjoy learning at the skills lab (Fig. 3).

**Table 5:** Mean competence scores of study participants in percentage by cumulative GPA

Skill station	CGPA < 3.25 mean score, (SD) a	CGPA > = 3.25 mean score, (SD) b	Mean score difference (c = a - b)	*p*-value (t-test)
Focused history taking	66.9 (15.3)	68.2(13.7)	−1.3	0.495
Precordium examination	71.4 (15.3)	76.3(16.1)	− 4.9	**0.024**
Interpreting chest x-ray & complete blood count for TB case	75.9(14.0)	81.0 (13.4)	− 5.1	**0.006**
Patient education & counseling for DM case	73.3 (13.8)	76.0 (14.0)	−2.7	0.144
Prescribing medications for malaria case	71.5 (17.3)	75.0(13.5)	− 3.5	0.120
Conduct Leopold’s maneuver	66.4 (15.3)	72.9 (17.5)	−6.5	**0.004**
MVA for incomplete abortion	60.6 (17.6)	64.6 (16.5)	−4.0	0.082
Managing childbirth	64.5(15.8)	67.6(16.5)	−3.1	0.173
Wound suturing	70.1(18.2)	80.5 (13.7)	−10.4	**< 0.001**
Emergency response for a polytrauma	72.6 (14.1)	77.8(13.1)	−5.1	**0.006**
Obtaining consent for hernia repair	79.6 (17.6)	83.4 (13.8)	−3.4	0.076
Performing LP	61.1 (17.9)	68.1(19.9)	−7.0	**0.007**
**Composite mean score**	**69.5 (7.3)**	**74.3 (6.9)**	−4.8	**< 0,001**

### Clinical practice exposure

**Fig. 4:**
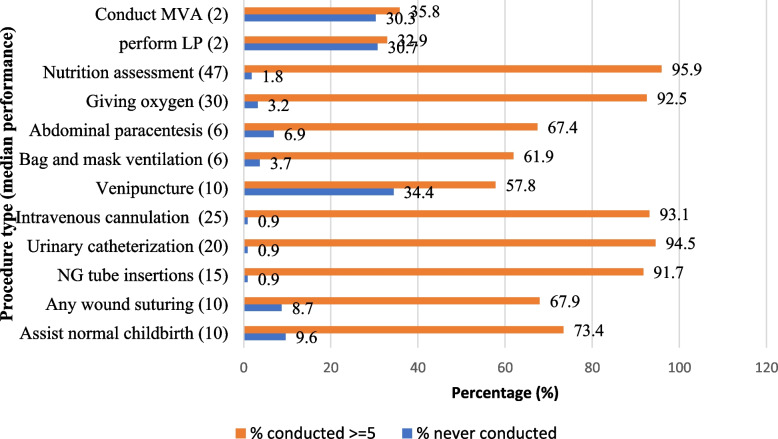
Percent of graduating medical students who conducted 5 or more procedures and never conducted

Of the 12 procedures assessed, the majority of students performed the following tasks more than five times: nutrition assessment (95.9%), urinary catheterization (94.5%), intravenous (IV) cannulation (93.1%), giving oxygen (92.5%), and nasogastric (NG) tube insertion (91.7%). On the contrary, significant proportions of students never performed venipuncture (34.4%), lumbar puncture (LP) (30.7%), manual vacuum aspiration (MVA) (30.3%), and assisted normal delivery (9.6%) (Fig. 4).

### Factors affecting competence scores of graduating medical students

Female medical students had 2.4% higher competence scores compared to their male counterparts (*p* = 0.03). On average. Medical students with a CGPA of < 3.00 had 7.1% lower competency scores compared to those with a CGPA of > 3.50 (*p* = 0.001). Similarly, students with a CGPA of 3.00–3.49 had on average 3.7% lower competency scores than those with a CGPA of > 3.50 (*p* = 0.001). For a unit increase in the satisfaction score of students’ motivations in the CLE, the mean competence score increased by 12.7% (*p* = 0.020) (Table 6).

**Table 6:** Bivariable and multivariable linear regression results to assess factors affecting the competence of graduating medical students

Independent variable	Bivariable model			Multivariable model		
	Unstandardized coefficient (B)	95% C.I. of B	*P*-value	Unstandardized coefficient (B)	95% C.I. of B	*P*-value
School type						
Public (ref)						
Private	−0.015	[− 0.056, 0.027]	.48	–	–	–
Sex						
Male (ref)						
Female	−.031	[−.062, 0]	**.05**	0.024	[0.003, 0.045]	**.03**
Age of student in years	0.014	[0.008, 0.019]	**<.001**	−0.005	[− 0.013, 0.003]	.19
Student entry scheme						
Graduate (reference)						
Direct	−.074	[−.101, −.046]	**<.001**	0.027	[−0.010, 0.064]	.15
CGPA						
> 3.50 (ref)	–	–	–	–	–	–
3–3.49	−0.004	[−0.024, 0.016]	.69	−0.037	[− 0.059, − 0.015]	**.001**
< 3.00	− 0.05	[− 0.072, − 0.028]	**<.001**	−0.071	[− 0.098, − 0.045]	**<.001**
Quality of simulation training	0.047	[−0.024, 0.117]	.19	−0.001	[− 0.071, 0.069]	.98
CLE Cases	0.492	[.418, .566]	**<.001**	−0.052	[−0.129, 0.026]	.19
CLE motivation	0.723	[0.636, .810]	**<.001**	0.127	[0.018, 0.235]	**.02**
CLE supervision	0.515	[.465, .566]	**<.001**	0.042	[−0.030, 0.114]	.25
CLE doctor-patient encounter	0.591	[.536, .647]	**<.001**	0.002	[−0.079, 0.084]	.95

The mean competence scores of students who performed tasks of giving oxygen above the median of 30, IV cannulations above the median of 25, urinary catheterization above the median of 20, and NG tube insertion above the median of 15 were significantly larger than that of students with task performance below the corresponding medians. (*p* < 0.031). Similarly, the mean competence scores of students who performed tasks of wound suturing more than the median of 10, venipuncture more than the median of 10, and abdominal paracentesis more than the median of 6 were significantly larger than that of students with task performance below the corresponding medians (*p* < 0.011) (Table 7)

**Table 7:** Mean competence difference of study participants by level of practice exposure (number of conducted procedures**)**

Conducted procedure	Competency score	Mean difference	95% CI	*P*-value
**Nutrition assessment**				
< or = 47	0.7077			
> 47	0.7253	−0.01767	−0.03748, 0.00214	0.08
**Giving oxygen**				
< or = 30	0.6951			
> 30	0.7343	−0.03917	− 0.05851, − 0.01981	**< 0.001**
**Intravenous cannulation**				
< or = 25	0.7044			
> 25	0.7267	−0.02225	−0.04234, − 0.00216	**0.03**
**Urinary catheterization**				
< or = 20	0.6996			
> 20	0.7313	−0.03171	− 0.05141, − 0.01202	**0.002**
**Nasogastric insertion**				
< or = 15	0.6989			
> 15	0.7304	−0.03152	− 0.05163, − 0.01141	**0.002**
**Assist normal delivery**				
< or = 10	0.7083			
> 10	0.7233	−0.01492	− 0.03526, 0.00542	0.07
**Conduct wound suturing**				
< or = 10	0.7042			
> 10	0.7279	−0.02362	−0.04354, − 0.00370	**0.01**
**Venipuncture**				
< or = 10	0.7015			
> 10	0.7296	−0.02809	−0.04783, − 0.00835	**0.005**
**Bag and mask ventilation**				
< or = 6	0.7094			
> 6	0.7235	−0.01413	−0.03399, 0.00573	0.162
**Abdominal paracentesis**				
< or = 6	0.6991			
> 6	0.7321	−0.03304	−0.05263, − 0.01345	**< 0.001**

## Discussion

After a successful primary healthcare expansion, Ethiopia has strengthened secondary and tertiary care aiming to increase its responsiveness to the population’s health needs. This progress has stimulated the rapid expansion of medical training in the country. With no congruent attention given to maintaining the training quality, the expansion has affected the medical schools in meeting the minimum pre-service education standards [28, 29]. Understanding the real effects of rapid training expansion and the challenges it poses is a critical step for improvement; particularly in contexts like Ethiopia where there is scanty evidence. To that end, we conducted this research to answer two main questions: what level of clinical competence did the undergraduate medical students master at the point of graduation? And which factors were associated with competence development?

The results of this study showed that the graduating medical students had suboptimal competence scores as a whole and in many competence areas as compared to the 75% cut-off score, signifying students’ capability gaps required for essential healthcare delivery. The pervasive shortages of experienced faculty and learning infrastructure, and challenging practical learning in Ethiopia’s medical schools coupled with the underdeveloped medical education regulation practices might be the underlying factors [7, 15, 30]. Comparable competence scores were also reported by studies conducted in Ethiopia and elsewhere [31–34]. Challenges of medical education due to shortages of critical training inputs and processes were similarly reported in Tanzania [35]. The competence gaps among the study participants made it clear that the medical graduates were not fully prepared for the responsibilities of general practitioners listed under the national scope of practice guidelines [20]. This means that the new graduates’ performance, confidence, professional identity, career progression, and quality of life can be affected [36, 37]. This all can have huge implications for the standards of patient care.

Effective preservice education for medical students requires high-quality clinical preceptorship and simulation training [38]. Repeated practice opportunities in clinical sites can boost the competencies learned and experiences acquired [39–41]. To that end, ensuring an optimal number and variety of cases in clinical settings is vital [42]. However; as stipulated in this study, performing hands-on clinical procedures by the medical students proved relatively more difficult. And a significant proportion of the students also had fewer practice exposures. On top of that, our study depicted that the medical students had challenging simulation and clinical learning environments. Studies conducted in other countries also discovered that the psychomotor abilities of final-year medical students were not fully developed [43–45]. The large number of enrolled students in Ethiopia’s medical schools might negatively affect the practical training in both simulation and clinical settings. Introducing medical education program accreditation and regulation has the potential to motivate schools to pursue quality [46]. Other researchers also corroborated our reports of the unnecessary effects of the rapid training scale-up and overwhelming students in Ethiopia [10–12, 29, 30]. However, many of the study participants had favorable perceptions regarding the number of students during practice. This might trigger questions about how well the schools used clinical rotations and scheduling to offset practice site overcrowding. And did the schools have adequate clinical sites used for student practice? [47]As per the findings of our study, the medical students’ motivation in clinical learning was associated with competency development. Unfortunately, the existing CLE gaps including the suboptimal availability of case varieties, motivation and supervision of students, and organization of clinical encounters affected the quality of student practice which might diminish the competence development [48–50].

Similar to what we reported, good academic performances were also associated with competence attainment in other studies [51]. This entails that medical schools should ensure that well-prepared students are enrolled and effectively taught, evaluated, and supported students across all stages of the curriculum. Despite many programmatic reports suggesting gender disparity in Ethiopia disfavoring females [52], this study depicted that female medical students had higher competence scores than males. They also had better scores in managing TB and malaria cases, conducting manual vacuum aspiration and lumbar puncture. Female nonphysician anesthesia students in Ethiopia similarly outperformed their male counterparts [53]. However, the male midwifery students had a better performance than the females [54].

### Strengths and weaknesses

Covering all the required clinical competency domains and considering all types of medical schools found in the country enabled us to generate high-quality evidence. We conducted a direct observation of student performance using OSCE tools which have acceptable reliabilities and high objectivity. The multiple quality indicators were evaluated in the causal chain of educational inputs, processes, and outcomes, providing a better picture of the training. To address logistical challenges, we widened the data collection period to include all schools as the academic calendars of medical schools were variable. The shortage of OSCE logistics was solved in collaboration with the medical schools. Since we did not get standardized assessment rubrics for our purpose, experts assisted in developing and piloting rubrics based on the curricula and standards.

## Conclusions

Medical students had suboptimal clinical competence. Lower competence scores were found in clinical procedures. A better CLE, higher cumulative GPA and academic performance, and more practice exposure were associated with high competence scores. We recommend that medical schools need to expand student clinical sites to primary healthcare units and private health facilities. Effective scheduling and clinical rotations are required to boost practice opportunities. Expanding and/or developing preceptors should be conducted. It is also imperative to address the simulation training gaps. Strengthening licensing examinations is also a way forward to ensure the graduates are fit for practice. Research studies are needed to understand the effects of the current medical education status on patient outcomes. Additional investigation is also required to assess the medical students’ ethics, leadership, communication, and collaboration skills.

## Data Availability

The datasets used and/or analyzed during the current study are available from the corresponding author upon reasonable request.

## Abbreviations

CBC: Complete blood count
CGPA: Cumulative grade point average
CLE: Clinical Learning Environment
CLEQ: Clinical Learning Environment Questionnaire
CPD: Continuing professional development
DM: Diabetes mellites
GRS: Global rating scale
IRB: Institutional Review Board
IV: Intravenous
LP: Lumbar puncture
MOE: Ministry of Education
MOH: Ministry of Health
MVA: Manual vacuum aspiration
NG tube: Nasogastric tube
OSCE: Objective structured clinical examination
SD: Standard deviation
SDL: Skills development laboratory
SOP: Scope of practice
SSA: Sub Sahara Africa
TB: Tuberculosis
USAID: United States Aids for International Development
WHO: World Health Organization
